# Of cattle and humans: A narrative review on the re-emergence of the New World screwworm (*Cochliomyia hominivorax*) and its threat to human health

**DOI:** 10.1371/journal.pntd.0014727

**Published:** 2026-09-21

**Authors:** Liesbeth Frias, Sergio Guerrero-Sanchez, Dirk U. Pfeiffer

**Affiliations:** 1 JCC Research Centre for Applied One Health Research and Policy Advice, City University of Hong Kong, Hong Kong Special Administrative Region of China; 2 Department of Infectious Diseases and Public Health, Jockey Club College of Veterinary Medicine and Life Sciences, City University of Hong Kong, Hong Kong Special Administrative Region of China; 3 Royal Veterinary College, London, England, United Kingdom; Hebrew University Hadassah Medical School, ISRAEL

## Abstract

Following more than two decades of successful elimination of the New World screwworm fly from Central and North America, new outbreaks emerged in Panama in 2023, initially affecting livestock, and rapidly spreading northward to impact not only livestock but also companion animals and humans. Forest degradation, increasing cattle density, and climate-driven shifts in the fly’s distribution—particularly its recolonization of regions it historically occupied—are converging to expose critical gaps in preparedness and cross-sector coordination. In this review, we examine the drivers of screwworm re‑emergence and present current clinical and epidemiological evidence of human infection, highlighting ongoing barriers to detection and response. Building on lessons from recent zoonotic health emergencies, we propose actionable One Health recommendations aimed at tackling the re-emergence of screwworm. An effective response will require sustained political commitment and coordinated cross-border collaboration to prevent further spread, protect vulnerable populations, and ensure that this myiasis is prioritized, ending the neglect of this tropical disease.

## Background

In July 2023, Panama declared a national zoo-sanitary emergency following a surge in New World screwworm (*Cochliomyia hominivorax*, Coquerel 1858) infestations among livestock. This outbreak rapidly spread to Costa Rica, Nicaragua, Honduras, and Guatemala in the following year [1,2]. By early 2025, screwworm cases initially confined to livestock began to be reported in companion animals and humans throughout Central America and southern Mexico, marking the first human and animal cases reported since screwworm was eliminated in 1991 [3–5].

The New World screwworm, also known as *screwworm*, *bicheira*, *bichera*, *gusanera,* or *gusano barrenador*, is an obligatory parasitic fly native to the Americas that develops in the living tissues of warm-blooded animals. Unlike other myiases (*i.e.*, parasitic infestations caused by fly larvae), screwworm is not self-limiting, can cause significant tissue damage, and may be fatal to the host [6]. The adult fly can lay up to 500 eggs at a time in an open wound, from which larvae emerge within the first 24 hours, burrowing into the wound and feeding on living tissue. Subsequent wound infections can ultimately lead to the host’s death [7].

Historically, the distribution of *C. hominivorax* originally extended from the southern United States to central Argentina, including the Caribbean [8]. The species was eliminated from North and Central America beginning in 1957 through the sterile insect technique, a biological control strategy that involved mass rearing, sterilization, and release of flies to disrupt reproduction [9]. At present, a dedicated facility in Panama is actively working to prevent the reintroduction of screwworm into Central and North America from South America, where the fly continues to pose a significant threat [10,11]. For over two decades, the region between Panama and the United States remained screwworm-free, underscoring the effectiveness of past elimination campaigns.

The recent resurgence of screwworm is closely linked to illegal cattle trafficking and ranching [12]. These illicit activities drive extensive deforestation across Central America, clearing forests for grazing and intensifying the livestock-wildlife interface [13–15]. The rise in screwworm cases in cattle reported in Panama in 2023 was further exacerbated by reduced monitoring during the coronavirus pandemic and supply chain disruptions [16]. These circumstances contributed to the breakdown of the invisible barrier at the Darien Gap, which had previously separated screwworm-free regions in the north from still infested areas in the south.

## Screwworm in a changing world: The effects of a warming climate and land use change

South America stands out not only for its substantial production and export of animal-derived products, but also for its globally significant biodiversity hotspots, such as the Amazon rainforest. However, the intensification and expansion of agricultural activities have posed a multifaceted challenge to environmental sustainability in the region [17–19]. As demand for animal products continues to rise, livestock density has increased markedly, with both direct and indirect environmental repercussions [20]. One of the most prominent mechanisms linking livestock density to ecological degradation is deforestation. Cattle ranching is the leading driver of forest loss in the Amazon, responsible for approximately 80% of deforestation in Brazil’s Amazon region [21,22]. Land is cleared either for direct grazing or to cultivate feed crops, resulting in habitat loss, reduced carbon sequestration, and ecosystem fragmentation [23]. For example, between August 2020 and July 2021, over 13,000 km^2^ of the Amazon rainforest were destroyed, primarily due to the expansion of pastureland for cattle [24,25].

The transformation of significant forest areas across Latin America intensifies the interface among wildlife, livestock, humans, and screwworm, creating conditions favorable to its survival and expansion. As forested areas are converted into pastures, wildlife is forced into smaller forest patches, increasing animal density and facilitating the infestation of the parasitic fly [26]. Moreover, the push for higher productivity in livestock systems often leads to increased cattle density [27], increasing the likelihood of contact between the fly and susceptible hosts, especially in resource-limited settings with poor or nonexistent biosecurity measures. The situation is further aggravated by the introduction of invasive species, which not only disrupt local ecosystems but also act as additional hosts for screwworm infestations. In Uruguay, for instance, feral pigs (*Sus scrofa*) have been reported with a screwworm prevalence of 4.36%, highlighting their significant role in maintaining this parasitic fly and increasing the risk of transmission to both wildlife and livestock [28]. Similar concerns have been raised in the United States, where expanding feral pig populations are viewed as potential reservoirs that could complicate surveillance, biosecurity, and eradication efforts if screwworm were to become established in wildlife [29].

Global warming can further shape the distribution and potential for expansion of the screwworm fly. Historically, colder, drier climates have limited the screwworm fly from establishing populations further north [30]. In contrast, warmer seasons have been linked to wider dispersal and higher incidence rates [31]. As the climate continues to warm, favorable conditions for screwworm survival and reproduction may persist year-round, enabling the species to expand its geographic range. Regional climate models predict a notable northward shift in the screwworm’s potential habitat, extending into the southern United States and reaching higher altitudes in Mexico [26,32]. Furthermore, warmer temperatures are anticipated to increase cattle tick populations, and their associated wounds, thereby increasing both the geographical spread of ticks and the risk of screwworm outbreaks [33].

To meet these challenges, it is critical to understand how weather influences the dynamics of the screwworm fly and the implications of climate change on its survival and reproduction. This would require the collection of robust biological and ecological data, including host distribution and density. Although considerable progress has been made in understanding the ecology of the screwworm fly (*e.g.*[12,26,32,34]), important knowledge gaps remain that constrain the development of models capable of accurately capturing its weather-driven biology, predicting its geographic spread, and being reliably applied across regions and emerging climate scenarios [35,36]. For example, the population structure of *C. hominivorax* across different spatial scales is still not fully resolved [37,38], and current information on thermal tolerance limits and temperature-dependent developmental rates is limited [39]. In addition, there is comparatively less ecological and epidemiological information for some key geographic areas, such as the Caribbean [40], further hindering model generalization. A more comprehensive understanding of screwworm ecology, integrating population genetics, climate sensitivity, and landscape-level processes, would substantially improve the development of robust risk assessments. These assessments could not only identify the factors driving screwworm establishment and spread, but also inform land-use and territorial planning strategies designed to mitigate risk and strengthen prevention and early-response efforts.

## Public health challenges: Understanding the impact of screwworm on human health

Although screwworm primarily affects livestock, it can also affect humans, causing significant morbidity and even mortality [41,42]. Like most myiases, screwworm infestation is often underestimated and regarded as minor [43,44]. Despite being zoonotic, the epidemiological impact of screwworm on human health remains challenging to evaluate. This difficulty arises because in most affected countries, myiases are still not designated as infectious diseases of obligatory notification, meaning there is no legal requirement to report cases to health authorities [45]. Consequently, available data are typically anecdotal, sporadic, and reliant on the informed views of specialized physicians and veterinarians. This high rate of underreporting makes it impossible to estimate credible indicators of morbidity, mortality, incidence, or prevalence, rendering screwworm a largely neglected disease, particularly in tropical and subtropical regions.

**Fig 1:**
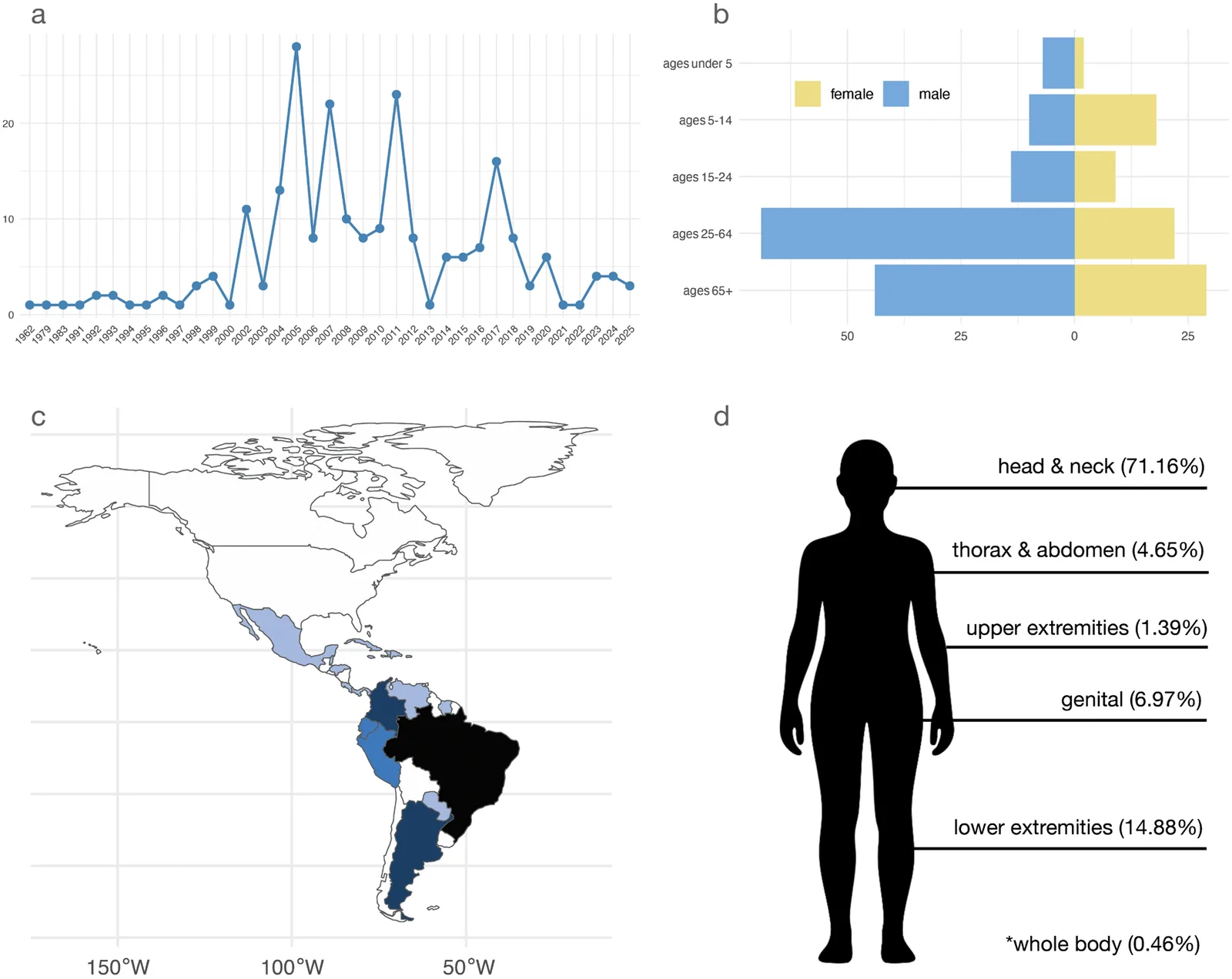
Documented cases of human infection with C. *hominivorax*, as reported in the literature. The figure illustrates **(a)** trends in reporting since 1962, **(b)** the demographic characteristics of affected individuals, **(c)** the geographic distribution of cases at the country level, and **(d)** the anatomical sites most commonly involved. Map generated in RStudio v.4.6.0 [46] using the rnaturalearth package, v.1.2.0 [47]; base vector data courtesy of Natural Earth (https://www.naturalearthdata.com/). The human silhouette is from PhyloPic (https://www.phylopic.org; T. Michael Keesey, 2026), created by Cagri CEVRIM under a CC0 1.0 Universal Public Domain Dedication.

Before the year 2000, most documented human cases in the scientific literature originated from countries in Central America, with a limited number of cases in Colombia and Ecuador (Fig 1A). As elimination campaigns rendered most Central American countries screwworm-free during the 1990s, literature from 2000 onward reports cases almost exclusively from South America, with only a few exceptions in the Caribbean. Notably, the majority of cases in South America were reported from Brazil (Fig 1C), where screwworm was identified across all regions, with the highest prevalence in the Southeast, particularly in Rio de Janeiro and São Paulo (63.2%) [48]. Argentina also reported several cases, indicating that *C. hominivorax* was distributed throughout most of the country, except for the arid and semi-arid regions [49,50]. No human cases were documented in Guyana or Bolivia, although these countries have been flagged as potential connection corridors for the fly in South America [34]. To date, Chile is the only country in the region that remains screwworm-free, likely protected by the natural geographic barriers of the world’s driest desert to the north and the Andes mountain range to the east, which have thus far shielded the country from tropical diseases affecting other parts of the continent. However, this assumption may soon be challenged by factors such as climate change and increased human and animal mobility [12,51].

Beyond its geographic spread, screwworm poses varying risks to different populations, influenced by age, health status, and living conditions. Parasitism is not uncommon in travelers returning from countries where screwworm is endemic [52,53]. Timely diagnosis, removal, and secure containment of all larvae, along with prompt reporting to public health and animal health authorities, are crucial for preventing the establishment and spread of myiasis-causing flies in areas where these do not currently occur [51,54]. Based on the identified case studies, most affected individuals were over the age of 25, with nearly twice as many cases reported in males compared to females (Fig 1B). Those most vulnerable tend to be very young or very old, ill or debilitated, and often have poor personal hygiene [55–58]. The incidence of myiasis was higher in rural areas, where the fly species responsible are more prevalent and where proximity to livestock and companion animals poses a risk to humans [59,60]. Individuals who cannot care for themselves were especially at risk, notably those with neurocognitive deficits, who were being artificially fed, or who were neglected [56,61]. Additional risk factors included alcoholism, homelessness, and specific predisposing medical conditions such as epilepsy and epidermal carcinoma [62–64]. Cases of screwworm were predominantly found on the scalp and naso-oropharyngeal region (Fig 1D). In children, infectious diseases such as chickenpox or pediculosis were a contributing factor for screwworm, particularly when they resulted in skin excoriation [65–67].

## Myiases, neglected among the neglected?

The World Health Organization prioritizes 20 neglected tropical diseases (NTDs), nearly all of which are infectious except for snakebite envenoming, and these diseases share several defining characteristics [68]. They disproportionately impact poor and marginalized populations, leading to substantial illness and death. They primarily affect communities in tropical and subtropical regions, where they often coexist with non-communicable diseases such as cardiovascular disease and diabetes, further complicating clinical outcomes and increasing the likelihood of poor health [69]. Importantly, NTDs are largely preventable and manageable through targeted public health interventions; however, they receive disproportionately limited attention in scientific research and funding compared with the significant health burdens they impose [68]. Although endemic zoonotic diseases such as myiases share many features with recognized NTDs, they are not officially included on the WHO’s list of NTDs. This exclusion contributes to their underrepresentation in both research and public health initiatives, resulting in less attention and fewer resources devoted to understanding and controlling these diseases, especially in a changing socioecological landscape. As a consequence, there is a notable lack of reliable data for estimating the prevalence and incidence of myiases, and limited insight into their impact on family economies or disability-adjusted life years (DALYs).

One major barrier to comprehensive data collection and effective intervention is stigma. Individuals suffering from myiases often face social stigmatization and discrimination due to the physical impairments and disfigurements associated with the condition [70]. Those with stigmatized diseases may delay seeking medical help to avoid social repercussions, which can worsen their condition and increase suffering [71]. Additionally, healthcare providers themselves may contribute to stigmatization, which can negatively impact service provision and reduce patients’ access to timely diagnosis and treatment [72]. Addressing stigma as a barrier to effective healthcare requires health education campaigns that develop information, education, and communication strategies resonating with all stakeholders. Such initiatives can help reduce stigma and social isolation for affected patients, improve treatment adherence, and foster community participation in control efforts [73]. Educational activities should be relatable and address real concerns, targeting not only patients but also their families, healthcare workers, and community leaders. Incorporating the perspectives of those directly affected can further enrich future research and intervention programs, ensuring a more holistic understanding of the barriers to effective care and promoting more inclusive solutions [74,75].

## Addressing the screwworm threat: Actionable recommendations for collaborative One Health management

The persistent challenge of screwworm infestation demands coordinated evidence-based action across human, animal, and environmental health sectors. Recent zoonotic outbreaks have demonstrated that siloed approaches are insufficient and that effective control hinges on the One Health framework, where surveillance, prevention, and response are integrated across disciplines and stakeholder groups [76,77]. However, commonly implemented reactive strategies, particularly the widespread use of insecticides and seasonal adjustments to livestock management practices such as dehorning, shearing, or calving, have shown limited long-term success [78]. Insecticide-based control, while still the most prevalent approach, is constrained by operational limitations. Probably the major unwanted consequence is the development of resistance in insects to components such as organophosphates [79,80] and pyrethroids [81]. Furthermore, due to the lack of specificity of available insecticides, their indiscriminate use has adverse consequences for ecosystems and the services they provide [82,83], not to mention the impact on human health [84]. Similarly, modifications to husbandry practices can directly affect farm productivity and profits, thereby reducing their adoption among livestock producers [78]. For example, it has been reported that there is a clear tension between implementing biosecurity regulatory measures, including quarantine, and the economic impact these strategies may have on production [85]. Changing the breeding schedule, as widely suggested, would require not only adjustments in logistics and manpower but also acceptance of additional risks, such as predation or malnutrition [78], not to mention a dramatic overhaul of the entire value chain.

These limitations highlight the need to move beyond predominantly reactive, top-down, and sector-specific interventions [86]. Strengthening transdisciplinary collaboration is central to these efforts. For instance, closer coordination between veterinary and public health services, as well as farmers organizations and ecologists could be operationalized through joint response teams that: (i) codesign and implement sustainable and practical surveillance programmes and coordinated outbreak response protocols; (ii) optimize resource use through shared infrastructure, logistics, and cross-training of personnel; (iii) assess the impact (both positive and negative), in a holistic manner, of the strategies and adapt accordingly; and (iv) expand access to services in rural and remote areas by integrating animal and human health outreach, fostering community trust, and promoting the uptake of preventive practices that are compatible with local production systems [74–77,87]. Together, these actions should provide a more realistic and context-sensitive pathway toward sustainable screwworm management under current ecological and socioeconomic conditions.

Community-based animal health workers (CAHWs) can have a substantial impact in this collaborative framework. They receive specialized training tailored to local animal disease risks and are equipped to provide preventive and curative services, including vaccination, castration, dehorning, and basic wound care [88]. Their close and ongoing engagement with animal owners and community leaders positions them to identify outbreaks, such as screwworm infestations, at an early stage, making them essential frontline reporters of cases in remote areas [89]. CAHWs also help raise awareness and encourage community participation in disease surveillance and response efforts. In areas with limited access to conventional health care, a partnership between rural doctors and traditional healers is essential to effectively manage screwworm infestations. Traditional healers not only bridge gaps between communities and health care institutions but also foster trust, strengthen resilience, and support communities during disease outbreaks and other public health challenges [90].

Case reporting matters. While myiases are not generally reported, they should be monitored in areas where screwworm is prevalent. Communities are often the first to observe changes in animal or environmental health that could indicate a disease outbreak [91]. Their ecological knowledge can complement scientific data, enhancing the effectiveness of early warning systems. Since animal cases are often detected before human cases, prompt reporting of these incidents can provide vital alerts to community health programs and facilitate a more rapid response. By integrating local observations with formal surveillance systems, communities can take an active role in identifying and responding to potential outbreaks. This collaborative approach not only enhances early detection and intervention but also builds trust between health officials and the public. As a result, cooperation and compliance are strengthened, ultimately improving the effectiveness of both animal and public health initiatives.

In areas where screwworm infestations have not been common for some time, it is imperative to educate new generations about the biology and life cycle of this parasite to facilitate effective detection and response efforts. Training in the prevention, detection, containment, and management of screwworm myiasis is essential, particularly in tropical areas, where eradication is complicated by the density of susceptible wildlife and their proximity to domestic animals and humans. At the community level, enhanced wound care for both humans and animals can help reduce fly populations and associated risks [92]. This is especially important for professionals in animal health, including those in private veterinary practices, governmental bodies, and individuals involved in wildlife monitoring, such as park rangers and ecologists. The effective surveillance and management of zoonotic diseases requires collaboration across multiple sectors, including human health, veterinary medicine, agriculture, education, wildlife management, environmental protection, and sanitation. Achieving coordinated action among these stakeholders presents significant challenges, particularly in aligning policies, priorities, resource allocation, and communication at national and international levels.

## Afterword

While this manuscript was under review, screwworm continued to expand across Mexico and toward the United States. By June 2026, the United States recorded its first screwworm case in nearly 60 years, while in August 2026, detections were reported in 30 of Mexico’s 32 states [93,94]. As of August 2026, there is a cumulative burden of over 209,400 animal cases and over 2,500 human cases [95]. Although cattle remain the most affected host species, the increasing proportion of cases in dogs in Mexico and Guatemala suggests that transmission is also becoming established in periurban and domestic settings, alongside continued spread through livestock production systems [93]. Human cases remain concentrated primarily in Mexico and Central America, while the only reported human case in the United States has been classified as imported or travel-associated [95]. In response, control measures have intensified through border closures and phased reopenings, quarantine measures, enhanced surveillance, and investments in sterile-fly production capacity in Texas (United States) and Chiapas (Mexico) [94,96].

## Methods

Our systematic review included articles on New World screwworm with a focus on human cases. We searched four academic literature databases: Scopus, Web of Science, Scielo, and the Biblioteca Virtual en Salud (BVS, “Virtual Library of Health”) for articles published up to the end of July 2025 with the terms: “screwworm” OR “*Cochliomyia hominivorax*”, AND “human cases” (Figs 1 and S1). Any screwworm case reported in humans with a parasitological confirmation of *C. hominivorax* (taxonomic or molecular) was eligible for inclusion. No language restrictions were applied, and articles in Spanish, Portuguese, French, and English were equally considered. Irrelevant articles identified by the search (*e.g.*, those containing the search terms but unrelated to New World screwworm cases in human patients) were discarded. Relevant articles were tallied for the following information: the patient’s sex and age category, the country where the case occurred, the site of infection, and risk factors associated with the case. Articles were excluded if they were population studies, where relevant individual information could not be retrieved. However, the reference lists of those articles were screened for relevant studies. From the assembled reference database, the authors independently reviewed the full-text articles of eligible papers and extracted relevant information using a predefined data extraction table. We additionally identified previous systematic reviews on screwworm and manually compiled relevant papers from these reviews [44,48,96,97].

Fig 1 was generated in RStudio v.4.6.0 [46] using a dataset of published human cases of *C. hominivorax*. Panel (a) shows annual numbers of reported cases since 1962, with pronounced peaks in 2005, 2007, and 2011. Panel (b) displays the age and sex distribution of cases, highlighting that males aged 25–64 account for the largest share. Panel (c) maps the country-level distribution of cases using the rnaturalearth package v.1.2.0 [47], with shading applied to countries with documented infections. Panel (d) illustrates the anatomical distribution of lesions by indicating the proportion of cases affecting each body region.

**Key learning points**

1. Following its re-emergence in livestock in Panama, the New World screwworm has spread rapidly northward, affecting companion animals and people.
2. Illegal cattle movements, expansion of cattle ranching, livestock intensification, and forest degradation are key drivers of re-emergence. Climate change may further expand screwworm habitat, increasing outbreak risk not only northward but also at higher elevations.
3. Neglected among the neglected: fragmented reporting and limited evidence on human cases across the region make it difficult to estimate the true burden and severity of human infestation.
4. Effective control requires integrated, community-based One Health action: stronger and more geographically extensive surveillance, timely case reporting, community engagement, adequately trained animal health workers, and coordination across the animal, human, and environmental health sectors.

**Key papers**

1. Zaldivar-Gomez A, Gomez-Vazquez JP, Iniesta-Valencia AJ, Figueroa-Martínez LG, Rico-Chávez O. Estimation the reinvasion of New World Screwworm (*Cochliomyia hominivorax*) in Central America: The role of animal movement in disease dispersal and control measures. Vet Parasitol Reg Stud Reports. 2025;59:101220.
2. Valdez-Espinoza UM, Fadda LA, Marques R, Osorio-Olvera L, Jiménez-García D, Lira-Noriega A. The reemergence of the New World screwworm and its potential distribution in North America. Sci Rep. 2025;15(1):23819.
3. George J, Häsler B, Mremi I, Sindato C, Mboera L, Rweyemamu M, et al. A systematic review on integration mechanisms in human and animal health surveillance systems with a view to addressing global health security threats. One Health Outlook. 2020;2(1):11.
4. Gutierrez AP, Ponti L, Arias PA. Deconstructing the eradication of New World screwworm in North America: retrospective analysis and climate warming effects. Med Vet Entomol. 2019;33(2):282–95.
5. Hall MJ, Wall RL, Stevens JR. Traumatic myiasis: a neglected disease in a changing world. Annu Rev Entomol. 2016;61(1):159–76.

## Supporting information

S1 Fig PRISMA flow diagram illustrating the systematic literature search and study selection. The chart summarizes study identification across traditional databases. A total of 1,226 records were initially identified, of which 1,145 were excluded after title and abstract screening, with reasons detailed in the diagram. In the end, 123 unique studies were included in the analysis.
(TIF)

S1 Data Studies included in this study. (DOCX)
