# Supplementary material for: Of cattle and humans: A narrative review on the re-emergence of the New World screwworm (Cochliomyia hominivorax) and its threat to human health
Source: PLoS Negl Trop Dis. 2026 Sep 21;20(9):e0014727. doi: 10.1371/journal.pntd.0014727 (PMC13592686; doi:10.1371/journal.pntd.0014727)
Supplement: Supplementary file 1 [file pntd.0014727.s002.docx]

**S1 Data. Studies included in this study.**

1. Africano FJ, Faccini-Martinez AA, Pérez CE, Espinal A, Bravo JS, Morales C. Pin-site myiasis caused by screwworm fly, Colombia. Emerg Infect Dis. 2015;21(5):905.
2. Akhoundi M, Mathieu A, Hannachi W, Nasrallah J, Quezel G, Blaizot R, Blanchet D, Ben Romdhane H, Epelboin L, Izri A. Morphological and molecular characterizations of *Cochliomyia hominivorax* (Diptera: Calliphoridae) larvae responsible for wound myiasis in French Guiana. Diagnostics. 2023;13(15):2575.
3. Alemán-Íñiguez JM, Reinoso-Quezada S. Infestación maxilar por larvas *Cochliomyia hominivorax*. Primer reporte ecuatoriano de debridación en miasis gingivo maxilar. REMCB. 2014;35(1):21–9.
4. Antunes AA, de Santana Santos T, Avelar RL, Neto EC, Neres BM, Laureano Filho JR. Oral and maxillofacial myiasis: a case series and literature review. Oral Surg Oral Med Oral Pathol Oral Radiol.2011;112(6):e81–5.
5. Batista-da-Silva JA, Borja GE, de Carvalho Queiroz MM. Miíases Humanas Causadas por Larvas de *Cochliomyia hominivorax* (Coquerel) (Diptera: Calliphoridae) em São Gonçalo, RJ, Brasil: Uma Abordagem Sócio-Econômica. EntomoBrasilis. 2011;4(3):144–6.
6. Batista-da-Silva JA, Borja GE, Queiroz MM. A severe case of cutaneous myiasis in São Gonçalo, Brazil, and a simple technique to extract New World screw-worm *Cochliomyia hominivorax* (Coquerel) (Diptera: Calliphoridae). Neotrop Entomol. 2012;41(4):341–2.
7. Cabrera F, Parra S, Palacios S. Miásis cutánea por *Dematobia hominis*. A propósito de 2 casos. Dermatol. 2002;11:32–5.
8. Calderón O, Sánchez C, Sandí J. Miasis oral por *Cochliomyia hominivorax* (Diptera: Calliphoridae) en una paciente geriátrica. Rev Cost Cienc Med. 1995;16(1,2):61–6.
9. Calderón O, Rivera P, Sánchez C, Solano M. *Cochliomyia hominivorax* (Diptera; calliphoridae) como agente causal de miasis aural en un niño de Costa Rica. Parasitol Dia. 1996:130–2.
10. Calderon HP, Rojas EC, Apt BW, Castillo OD. Cutaneous myiasis due to *Cochliomyia hominivorax* associated with seborrheic dermatitis. Rev Med Chile. 2017;145(2):250–4.
11. Calderon-Castrat X, Idrogo-Bustamante JL, Peceros-Escalante J, Ballona R. Wound myiasis caused by *Cochliomyia hominivorax*: the role of entodermoscopy. Int J Dermatol. 2017;56(3):330–2.
12. Casas Pérez L, Martín Martínez JG, Noa Barrios A. Caso de miasis orbitaria. Arch Med Camagüey. 2009;13(5):0–0.
13. Castro-Hidalgo J, Nuñez NM, Igreda AN, Velit JZ. Cutaneous myiasis by *Cochliomyia hominivorax* Coquerel, 1858 (Diptera: Calliphoridae) associated with oral neoplasia, in geriatric patient. Neotrop Helminthol. 2018;12(2):233–42.
14. Cazorla-Perfetti DJ, Acosta-Quintero ME, Morales P, Bermúdez SE, Rodriguez-Morales AJ. Pin-site myiasis: an emerging infectious complication of external bone fixation? Int J Infect Dis. 2009;13(6):e514–6.
15. Cedeño JA, Keil C, Ocampo JE, Zapata M, Guerrero M. Miasis auricular por *Cochliomyia hominovorax*. Rev Fac Cien Med (Quito). 2014;39(2):87–90.
16. Chicarelli M, Daniel AN, Santoro MA, Teodoro U. Miíase humana bucal por *Cochliomyia hominivorax* (Coquerel, 1858) em Nova Esperança, estado do Paraná, Brasil. RFO-UPF. 2002;7(2).
17. Chico M, Córdova M, Calvopiña M, Guderian R. Miasis humana en el Ecuador. Rev Médica Vozandes. 1994;3:101–8.
18. Clyti E, Couppie P, Cazanave C, Fouque F, Sainte-Marie D, Pradinaud R. Traitement des myiases dues à *Cochliomyia hominivorax* par application locale d’ivermectine. Bull Soc Pathol Exot. 2003;96:410–1.
19. Costa DC, de Tarso Ponte Pierre-Filho P, Mac Cord Medina F, Mota RG, Carrera CR. Use of oral ivermectin in a patient with destructive rhino-orbital myiasis. Eye. 2005;19(9):1018–20.
20. Costa FS, Bellotti A, Farah GJ, Camarini ET. Hipertratamento de miíase decorrente de trauma facial complexo. Rev Cir Traumatol Buco-Maxilo-Fac. 2012;12(3):25–30.
21. Couppié P, Roussel M, Rabarison P, Sockeel MJ, Sainte‐Marie D, Marty C, Carme B. Nosocomial nasal myiasis owing to *Cochliomyia hominivorax*: a case in French Guiana. Int J Dermatol. 2005;44(4):302–3.
22. da Silva BB, Borges US, Pimentel IC. Human vaginal myiasis caused by *Cochliomyia hominivorax*. Int J Gynecol Obstet. 2005;89(2):152–3.
23. da Silva BB, Vieira SC, Coelho EG, Machado V, Borges US, da Silveira Filho MA. Genital myiasis in a woman with psychiatric disturbance. BJOG. 2005;112(7):1000–1.
24. de Araújo RJ, Corrêa AM, Santos WR, Júnior MT. Advanced stage of oral myiasis in children: a clinical case report. Quintessence Int. 2008;39(1):39–43.
25. de Arruda JA, de Oliveira Silva LV, Silva PU, de Figueiredo EL, Callou G, Mesquita RA, do Egito Vasconcelos BC. Head and neck myiasis: a case series and review of the literature. Oral Surg Oral Med Oral Pathol Oral Radiol. 2017;124(5):e249–56.
26. de Kaminsky RG. Nosocomial myiasis by *Cochliomyia hominivorax* in Honduras. Trans R Soc Trop Med Hyg. 1993;87(2):199–200.
27. de la Ossa N, Castro LE, Visbal L, Santos AM, Díaz E, Romero-Vivas CM. Miasis cutánea por *Cochliomyia hominivorax* (Coquerel) (Díptera: Calliphoridae) en el Hospital Universidad del Norte, Soledad, Atlántico. Biomédica. 2009;29(1):12–7.
28. de Román EM, García ME, Quijada J, Marcial T. Un caso urbano de miiasis cutánea humana. Entomotropica. 2004;19(1):49–50.
29. de Souza Barbosa T, Salvitti Sá Rocha RA, Guirado CG, Rocha FJ, Graduate student, Duarte Gavião MB. Oral infection by Diptera larvae in children: a case report. Int J Dermatol. 2008;47(7):696–9.
30. Devoto MH, Zaffaroni MC. Orbital myiasis in a patient with a chronically exposed hydroxyapatite implant. Ophthal Plast Reconstr Surg. 2004;20(5):395–6.
31. Duque CS, Mosquera CA, Casiano RR, Abreu CE. Radiologic findings in sinonasal myiasis. Otolaryngol Head Neck Surg. 2006;135(4):638–9.
32. Duque FL, Ardila CM. Oral myiasis caused by the screwworm *Cochliomyia hominivorax* treated with subcutaneous ivermectin and creolin: report of six cases after trauma. Dent Traumatol. 2011;27(5):404–7.
33. Durand B, Sänger I, Santillán Iturres M. A case of myiasis in stage IV pelvic organ prolapse. Int Urogynecol J. 2023;34(1):313–5.
34. Durão C, Barros A, Campos P. A rare case of digital myiasis. J Infect Public Health. 2017;10(6):886–7.
35. Duro EA, Mariluis JC, Mulieri PR. Umbilical myiasis in a human newborn. J Perinatol. 2007;27(4):250–1.
36. Enríquez JD, Rosillo JC, Cuzco CC, Rodríguez-Hidalgo R, Calvopina M. Miasis orbital severa causada por *Cochliomyia hominivorax* en la región andina de Ecuador. Rev Mex Oftalmol. 2016;90(1):43–7.
37. Escandón-Vargas K, Cabezas F, Díaz CJ. An unusual case of Darier disease complicated with a parasitic infestation. SKINmed. 2015;13(2):142–4.
38. Espinoza A, Quiñones-Silva J, Garay O. Myasis in oral cavity by *Cochliomyia hominivorax*: a case report. Rev Peru Med Exp Salud Publica. 2009;26(4):573–6
39. Failoc-Rojas VE, Silva-Díaz H. Review of cases and a patient report of myiasis with tracheostomy, Peru. Emerg Infect Dis. 2016;22(3):563.
40. Failoc-Rojas VE, Molina-Ayasta C, Salazar-Zuloeta J, Samamé A, Silva-Díaz H. Case report: myiasis due to *Cochliomyia hominivorax* and *Dermatobia hominis*: clinical and pathological differences between two species in Northern Peru. Am J Trop Med Hyg. 2017;98(1):150.
41. Feka-Homsy P, L’Huillier AG, Monod L, Monin E, Guinand N, Schwob JM. *Cochliomyia hominivorax* aural myiasis in a 7-year-old traveler. IDCases. 2025:e02327.
42. Fernández RE, García TB, Borroto AC, de Armas RA. Miiasis facial por gusano barrenador del ganado asociado a un carcinoma. Presentación de dos pacientes. Medicentro Electrónica. 2010;14(4).
43. Fernández LM, Romay AA, Burgos FG, Ordaz LM, Figueroa LL. Miasis cutánea asociada a carcinoma basoescamoso: reporte de un caso por *Cochliomyia hominivorax*. Dermat Cosmet Méd Quir. 2020;17(4):284–7.
44. Figueiredo RR, Dorf S, Couri MS, Azevedo AA, Mossumez F. Corpos estranhos animados em otorrinolaringologia. Rev Bras Otorrinolaringol. 2002;68:722–8.
45. Fox I, Galindo L. Human Cutaneous Myiasis due to the Primary Screw-Worm *Callitroga hominivorax* (Coquerel) in Puerto Rico. Am J Trop Med Hyg. 1962;11:96–7.
46. Gealh WC, Ferreira GM, Farah GJ, Teodoro U, Camarini ET. Treatment of oral myiasis caused by *Cochliomyia hominivorax*: two cases treated with ivermectin. Br J Oral Maxillofac Surg. 2009;47(1):23–6.
47. Gomez RS, Perdigão PF, Pimenta FJ, Leite AR, de Lacerda JT, Neto AC. Oral myiasis by screwworm *Cochliomyia hominivorax*. Br J Oral Maxillofac Surg. 2003;41(2):115–6.
48. Gontijo JR, Bittencourt FV. Wound myiasis: the role of entodermoscopy. An Bras Dermatol. 2018;93(5):746–8.
49. González C, Salamanca JC, Olano V, Pérez CE. Miasis cavitaria: reporte de un caso. Revista Med. 2008;16(1):95–8.
50. Guanga I, Cruz C. Miasis nasal. Cambios. 2006;5:317–9
51. Gutierrez A. Nosocomial oral myiasis in an intensive care unit. An Fac Med. 2019;80(3):354–7.
52. Hernández RV, Asturias MÁ. *Cochliomyia hominivorax* como agente de miasis en humanos en Medellín, Colombia. In: Memorias XXI Congreso Panamericano de Ciencias Veterinarias, Guadalajara, Jalisco, México. 2008.
53. Jorge IF, Santana LR, Silveira MA, Brunetta DM, Kaufman J, Barroso‐Duarte F. Oro-nasal myiasis in a lymphoma patient. Br J Haematol. 2016;175(5):757.
54. Júnior SM, Asprino L, Prado ÂP, Moreira RW, de Moraes M. Oral myiasis caused by *Cochliomyia hominivorax* treated nonsurgically with nitrofurazone: report of 2 cases. Oral Surg Oral Med Oral Pathol Oral Radiol Endod. 2010;109(3):e70–3.
55. Konkol KA, Longfield RN, Powers NR, Mehr Z. Wound myiasis caused by *Cochliomyia hominivorax*. Clin Infect Dis. 1992;14(1):366.
56. Kron MA. Human infestation with *Cochliomyia hominivorax*, the New World screwworm. J Am Acad Dermatol. 1992;27:264–5.
57. Laureano Filho JR, Bezerra TP, Lima FT, Campelo RI. Extensive oral lesion colonized with 601 myiasis larvae. Trop Doct. 2011;41(1):61–2.
58. Levi Duque Serna F, Valderrama Hernández R, González Robledo JA. Tratamiento de miasis oral con ivermetina: notificación de tres casos causados por *Cochliomyia hominivorax* (Coquerel). Rev Fac Odontol Univ Antioq. 1998:41–7.
59. Lindsay R, Stancil J, Ray JM. Myiasis of facial wounds by *Cochliomyia hominivorax* sustained in a natural disaster in Haiti. Otolaryngol Head Neck Surg. 2010;143(4):595–6.
60. Lopes-Costa PV, dos Santos AR, Pereira-Filho JD, Da Silva BB. Myiasis in the uterine cavity of an elderly woman with a complete uterine prolapse. Trans R Soc Trop Med Hyg. 2008;102(10):1058–60.
61. Magistrello P, Avellaneda M, Curutchet ML, Belmont Wasserlaufe, Krause M, Torillo EL. Miasis ocular y bucal, a propósito de dos casos. Ludovica Pediátr. 2023:39–45.
62. Mariwalla K, Langhan M, Welch KA, Kaplan DH. Cutaneous myiasis associated with scalp psoriasis. J Am Acad Dermatol*.* 2007;57(2):S51–2.
63. Martinez CA, Romani G, Priolli DG, Campos AA, Carneiro VD, Dalbem CA. Miíase vulvar: relato de caso. Rev Bras Ginecol Obstet. 2003;25:291–5.
64. Martins LG, Barbosa TM, Gama RA. Myiasis in humans: case reports in Northeastern Brazil including multispecies co-infestation by Sarcophagidae. Parasitol Int. 2021;85:102436.
65. Massa AF, Mata RR, Hernández CS. Report of case series of genitourinary myiasis in Mexico. Rev Mex Urol. 2025;84(5):1–1.
66. Mehr Z, Powers NR, Konkol KA. Myiasis in a wounded soldier returning from Panama. J Med Entomol. 1991;28(4):553–4.
67. Mendivil JA, El Shammaa NA. Aural myiasis caused by *Cochliomyia hominivorax*: case report. Mil Med. 1979;144(4):261–2.
68. Menghi CI, Gatta CL, Oliva A. Otomiasis por *Cochliomyia hominivorax* en dos niños del conurbano bonaerense, Argentina. Rev Argent Microbiol. 2010;42(3):176–8.
69. Menghi CI, Arias LE, Gatta CL, Perazzo E, Dorronzoro M, Turlan N, Martínez E, Vay C. Miasis por *Cochliomyia hominivorax* en la Argentina. Medicina (Buenos Aires). 2020;80(2):185–8.
70. Miranda H. Miasis en Trujillo, Perú: observaciones clínicas y entomológicas. Folia Dermatol Peru. 2007;18(1):13–7.
71. Moissant de Román E, García ME, Quijada J, Simoes D, Marcial T. Miasis cutánea humana. Un caso clínico. Kasmera. 2004;32(1):12–5.
72. Neto CA, Monnazzi MS. Oral myiasis in a patient with neurological signs. Br J Oral Maxillofac Surg. 2016;54:1146–7.
73. Novo-Neto JP, Santos FD, Pontes AE, Ribeiro FS, Scannavino FL, Martins AT. Oral myiasis caused by *Cochliomyia hominivorax* in a disabled person. Case Rep Pathol. 2015;904658.
74. Oliveira JA, Machado MI, Oliveira MP. Miíase de língua: relato de um caso de infestação hospitalar. Rev Cir Traumatol Buco-Maxilo-Fac Camaragibe. 2008;8(4):47–50.
75. Osorio J, Moncada L, Molano A, Valderrama S, Gualtero S, Franco-Paredes C. Role of ivermectin in the treatment of severe orbital myiasis due to *Cochliomyia hominivorax*. Clin Infect Dis. 2006;43(6):e579.
76. Osorio JH. Diagnostic mistake and wrong treatment of cutaneous myiasis by *Cochliomyia hominivorax* (Coquerel)(Diptera: Calliphoridae). Braz J Biol Sci. 2016;3(5):e459-e459.
77. Paris LA, Viscarret M, Uban C, Vargas J, Rodriguez-Morales AJ. Pin-site myiasis: a rare complication of a treated open fracture of tibia. Surg Infect. 2008;9(3):403–6.
78. Passos MR, Varella RQ, Tavares RR, Barreto NA, Santos CC, Pinheiro VM, Bravo RS, Morelhi MH. Vulvar myiasis during pregnancy. Infect Dis Obstet Gynecol. 2002;10(3):153–8.
79. Pasternak J, Joo SH, Ganc AJ, Junior MD, Morsh RD, Pinto TH. A case of throat *Cochliomyia hominovorax* infestation. Einstein. 2007;5(2):170–2.
80. Paz LE, Morales FÁ, Llambía FÁ. Presentación de un caso de miasis cervical por gusano barrenador. Mediciego. 2004;10(1).
81. Pierre-filho PD, Minguini N, Pierre LM, Pierre AM. Use of ivermectin in the treatment of orbital myiasis caused by *Cochliomyia hominivorax*. Scand J Infect Dis. 2004;36(6-7):503–5.
82. Pinos VH, Ortiz-Prado E, Bermeo C, Leon J, Armijos L, Almeida E. A unique case of facial burn superinfected with *Dermatobia hominis* larvae resulting in a bilateral enucleation of the eyes. Trop Doct. 2014;44(4):235–7.
83. Piña-Tornés AA, Salvador-Fernández CL, Lindao-Camacho R, González-Longoria-Boada LB, Vintimilla-Burgos NP, Selles Almarales M. Massive cutaneous myiasis mimicking brain invasion. Case report and literature review. Medicas UIS. 2016;29(2):145–53.
84. Powers NR, Yorgensen ML, Rumm PD, Souffront W. Myiasis in humans: an overview and a report of two cases in the Republic of Panama. Mil Med. 1996;161(8):495–7.
85. Ramírez Rivera JI, Mieles Figueroa JM, Penafiel Vicuña CI, Ramírez Rivera ME. Miasis en la colostomía de un lactante. Rev Chil Cir. 2017;69(4):281–2.
86. Rawlins SC, Barnett DB. Internal human myiasis. West Indian Med J. 1983;32(3):184–6.
87. Reinoso-Quezada S, Alemán-Iñiguez JM. Rara miasis maxilar por *Cochliomyia hominivorax*: reporte de caso, actualidad y entomología. Rev Esp Cir Oral Maxilofac. 2016;38(2):111–6.
88. Ribeiro AL, de Almeida TE, Júnior JS, de Araújo Castro JF, Pinheiro JD. Oral myiasis: does an indication for surgical treatment still exist? Two case reports. Oral Surg Oral Med Oral Pathol Oral Radiol*.* 2012;114(3):e10–4.
89. Rodrigues FT, Klemig LR, Cardozo MR, Alves PC, Aguiar VM, Lessa CS. Myiasis associated with an invasive ductal carcinoma of the left breast: case study. Rev Inst Med Trop São Paulo. 2017;59:e35.
90. Saraiva VD, Amaro MH, Belfort Jr R, Burnier Jr MN. A case of anterior internal ophthalmomyiasis: case report. Arq Bras Oftalmol. 2006;69:741–3.
91. Shinohara EH, Martini MZ, Oliveira Neto HG, Takahashi A. Oral myiasis treated with ivermectin: case report. Braz Dent J. 2004;15:79–81.
92. Shoaib A, McCall PJ, Goyal R, Perkins HL, Richmond WD. First urological presentation of New World screw worm (*Cochliomyia hominivorax*) myiasis in the UK. BJU Int. 2000;86:402.
93. Sweis IE, Griffith HB, Pensler JM. Souvenirs from Belize: the botfly and the screwworm fly. Plast Reconstr Surg. 1997;99(3):868–70.
94. Tai R, Marsh MA, Rao R, Kurniali PC, DiNino E, Meharg JV. Nasal myiasis caused by *Cochliomyia hominivorax* in the United States: a case report. Am J Infect Dis. 2011;7(4):107.
95. Takahagi RU, Gonçalves FP, Madeira NG, Schellini SA. Oftalmomiíase externa causada por *Cochliomyia hominivorax*. Rev Bras Oftalmol. 2018;77(4):219–21.
96. Teixeira WF, Cruz BC, Maciel WG, FELIPELLI G, Matos LV, Costa AJ. Miíase cervical em humano provocada por larvas de *Cochliomyia hominivorax* no estado de Minas Gerais, Brasil–relato de caso. Ars Vet. 2013;29(4):110.
97. Theppote A, Laborde Y, Knoepp L, Thomas S, Nnedu ON. Cutaneous Myiasis in Rural Haiti. Ochsner J. 2020;20(3):331–3.
98. Thyssen PJ, Nassu MP, Costella AM, Costella ML. Record of oral myiasis by *Cochliomyia hominivorax* (Diptera: Calliphoridae): case evidencing negligence in the treatment of incapable. Parasitol Res. 2012;111(2):957–9.
99. Tortolero LL, Cazorla-Perfetti D, Moreno PM. Caso de miiasis orbitaria severa humana por *Cochliomyia hominivorax* (Diptera: Calliphoridae) asociada con carcinoma espinocelular en el estado Falcón, Venezuela. Saber. 2017;1(29).
100. Trombetta L, Bava J. Cutaneous myiasis due to *Cochliomyia hominivorax* in a drug user. J Infect Dev Ctries. 2009;3(11):873–6.
101. Venegas-Montero DP, Alfaro-Vellanero MJ, Rojas-Araya D, Calderón-Arguedas Ó, Vargas-Castro CM, Baldioceda-Villarreal A, Chaves-González LE, Camacho-Leandro J, Troyo A. Case report: Re-emergence of *Cochliomyia hominivorax* in Costa Rica: report of a human myiasis case 23 years after elimination. Am J Trop Med Hyg. 2024;111(5):1020.
102. Victoria J, Trujillo R, Barreto M, Msph. Myiasis: a successful treatment with topical ivermectin. Int J Dermatol. 1999;38(2):142–4.
103. Villamil-Gómez WE, Cardona-Ospina JA, Prado-Ojeda JS, Hernández-Prado H, Figueroa M, Causil-Morales PN, Pérez-Reyes K, Palechor-Ocampo LA, Rodríguez-Morales AJ. Pin-Site myiasis caused by screwworm fly in nonhealed wound, Colombia. Emerg Infect Dis. 2019;25(2):379.
104. Visciarelli E, Costamagna S, Lucchi L, Basabe N. Miasis humana en Bahía Blanca, Argentina. Periodo. 2000;2005:605–11.
105. Yang LJ, Abramovici G, Brieva J, Paller AS. A “boring” worm? New World screwworm neuromyiasis in a patient with Darier disease. JAAD Case Rep. 2025;59:50–2.
106. Zupan-Kajcovski B, Simonian H, Keller JJ, Faber WR. Cutaneous myiasis caused by a double infestation with larvae of *Dermatobia hominis* and *Cochliomyia hominivorax*. Ned Tijdschr Geneeskd. 2004;148(42):2086–9.
